# Safety and efficacy of lenvatinib by starting dose based on body weight in patients with unresectable hepatocellular carcinoma in REFLECT

**DOI:** 10.1007/s00535-021-01785-0

**Published:** 2021-05-04

**Authors:** Takuji Okusaka, Kenji Ikeda, Masatoshi Kudo, Richard Finn, Shukui Qin, Kwang-Hyub Han, Ann-Lii Cheng, Fabio Piscaglia, Masahiro Kobayashi, Max Sung, Minshan Chen, Lucjan Wyrwicz, Jung-Hwan Yoon, Zhenggang Ren, Kalgi Mody, Corina Dutcus, Toshiyuki Tamai, Min Ren, Seiichi Hayato, Hiromitsu Kumada

**Affiliations:** 1 National Cancer Center Hospital, Tokyo, Japan; 2 Toranomon Hospital, Tokyo, Japan; 3 Faculty of Medicine, Kindai University, Osaka, Japan; 4 Geffen School of Medicine, UCLA Medical Center, Santa Monica, CA USA; 5 Nanjing Bayi Hospital, Nanjing, Jiangsu China; 6 Severance Hospital, Yonsei University, Seoul, Republic of Korea; 7 National Taiwan University Hospital, Taipei, Taiwan; 8 IRCCS Azienda Ospedaliero-Universitaria di Bologna, Bologna, Italy; 9 Tisch Cancer Institute at Mount Sinai, New York, NY USA; 10 Sun Yat-Sen University Cancer Center, Guangzhou, China; 11 Narodowy Instytut Onkologii, Warsaw, Poland; 12 Seoul National University Hospital, Seoul, Republic of Korea; 13 Zhongshan Hospital Fudan University, Shanghai, China; 14 Eisai Inc., Woodcliff Lake, NJ USA; 15 Eisai Co. Ltd, Tokyo, Japan

**Keywords:** REFLECT, Lenvatinib, Hepatocellular carcinoma, Body weight, Dosing

## Abstract

**Background:**

REFLECT was an open-label, phase 3 study comparing the efficacy and safety of lenvatinib versus sorafenib in patients with unresectable hepatocellular carcinoma (uHCC). Based on phase 2 study (Study 202) results, body weight-based dosing for lenvatinib was used in REFLECT to minimize dose disruptions and modifications needed to address dose-related adverse events. This post hoc analysis of REFLECT data assessed lenvatinib efficacy and safety by body weight group.

**Methods:**

The study randomly administered lenvatinib (*n* = 476) or sorafenib (*n* = 475) to patients with untreated (no prior systemic therapy) uHCC. Lenvatinib starting-dose data were stratified by body weight: patients weighing < 60 kg received 8 mg/day; patients weighing ≥ 60 kg received 12 mg/day. Overall survival (OS), progression-free survival (PFS), objective response rate, and safety were assessed.

**Results:**

Survival outcomes and safety profiles appeared similar between the two body-weight-based lenvatinib starting-dose groups. Median OS for patients in the < 60 kg body weight group (*n* = 153) was 13.4 months [95% confidence interval (CI) 10.5–15.7] compared to 13.7 months (95% CI 12.0–15.6) in the ≥ 60 kg body weight group (*n* = 325). In both lenvatinib groups, PFS was 7.4 months (< 60 kg group: 95% CI 5.4–9.2; ≥ 60 kg group: 95% CI 6.9–9.0). Treatment-emergent adverse events (TEAEs) required dose modifications in 43.0% in the < 60 kg body weight group and 57.5% in the ≥ 60 kg body weight group.

**Conclusions:**

This exploratory analysis of data from REFLECT indicated that body weight-based lenvatinib dosing in patients with uHCC was successful in maintaining efficacy, with comparable rates of TEAEs and dose modifications in the two body weight groups.

**Clinincal trial:**

Trial registration ID: ClinicalTrials.gov # NCT01761266

**Supplementary Information:**

The online version contains supplementary material available at 10.1007/s00535-021-01785-0.

## Introduction

Hepatocellular carcinoma (HCC) is a major global cause of cancer-related deaths [1, 2]. The worldwide estimate of liver cancer mortality in 2018 was 8.2%, equating to approximately 780,000 deaths [1]. Unfortunately, most patients will eventually reach a stage of HCC when potentially curative therapies such as resection or transplantation are no longer clinically indicated [3].

At the time of the REFLECT study, sorafenib was the only approved first-line systemic treatment for HCC, but the approval of lenvatinib provided another option [2, 4, 5]; more recently atezolizumab in combination with bevacizumab became another front-line option [6]. Lenvatinib is a multitargeted tyrosine kinase inhibitor of vascular endothelial growth factor receptors 1–3, fibroblast growth factor receptors 1–4, platelet-derived growth factor receptor *α*, RET, and KIT [7–10]. Lenvatinib monotherapy is approved for the treatment of patients with locally recurrent or metastatic, progressive, radioactive-iodine-refractory, differentiated thyroid cancer, and for the first-line treatment of patients with unresectable HCC (uHCC) [4]. Lenvatinib is metabolized in the liver, and impaired hepatic function in patients with uHCC may impact lenvatinib metabolism; as such, dose adjustments based on weight are required for this patient population [11].

Diseases of the liver pose a unique dilemma for dose determination because many drugs are metabolized in the liver, and impaired hepatic function can impact pharmacokinetic parameters. As specific measures do not currently exist to estimate to what degree hepatic impairment might affect these pharmacokinetic parameters, clinical studies in patients with hepatic impairment are valuable in determining appropriate doses for this vulnerable population [12]. The US Food and Drug Administration recommends that a pharmacokinetic study be conducted in patients with hepatic impairment if hepatic metabolism and/or excretion pathways account for a substantial portion of the elimination process of a parent drug or active metabolite, or if the drug has a narrow therapeutic range [12].

Previously, the phase 2 Study 202 (NCT00946153) examined the efficacy and safety of lenvatinib in Asian patients (*N* = 46) with HCC [13]. No differences have been found in the metabolism of lenvatinib in Asian versus non-Asian populations [14]. Although lenvatinib displayed promising antitumor activity at the 12 mg/day dose, most patients (74%) required a dose reduction to address increased rates of adverse events. A correlation was found between lower body weight and early study drug discontinuation or a need for dose reduction of lenvatinib [13]. Pharmacokinetic results and exposure–response analyses of data from patients with HCC who were enrolled in Study 202 showed that lower body weight was associated with greater lenvatinib area under the plasma concentration–time curve (AUC), and with dose reduction or earlier treatment discontinuation [15]. Specifically, a relationship was observed between treatment-emergent adverse events (TEAEs) leading to dose reduction or discontinuation and baseline body weight [15]. Taken together, data from these pharmacokinetic simulations suggested a body weight-based dose regimen in patients with HCC of lenvatinib 8 mg daily for patients weighing < 60 kg and lenvatinib 12 mg daily for patients weighing ≥ 60 kg [15].

This body-weight-based lenvatinib dose regimen was used in REFLECT, a phase 3 study that assessed lenvatinib as first-line therapy for patients with uHCC. In REFLECT, lenvatinib treatment met the primary endpoint of noninferiority to sorafenib in terms of overall survival (OS) [16]. In addition, lenvatinib treatment resulted in statistically significant improvements in secondary endpoints (ie, PFS and ORR) compared with sorafenib.

In this exploratory post hoc analysis, efficacy and safety outcomes from the randomized phase 3 REFLECT trial were analyzed by the lenvatinib starting dose, based on patients’ body weights.

## Methods

### Study design

REFLECT was an international, randomized, phase 3, multicenter, open-label, noninferiority trial in patients with uHCC [16]. Full details of the study design and methodology for REFLECT have been reported previously (ClinicalTrials.gov number: NCT02501096) [16]. This post hoc analysis focused on comparing safety and efficacy outcomes between the two body weight-based lenvatinib dosing groups.

### Patients

Briefly, 954 patients were randomly assigned using a 1:1 ratio to receive either lenvatinib or sorafenib in 28-day cycles. The starting dose for lenvatinib was based on body weight: patients weighing ≥ 60 kg (higher body-weight group) received lenvatinib 12 mg/day and patients weighing < 60 kg (lower body-weight group) received lenvatinib 8 mg/day. Patients randomly assigned to sorafenib received 400 mg twice daily, with no weight stratification.

Eligible patients were ≥ 18 years of age, had histologically or cytologically confirmed uHCC, ≥ 1 measurable target lesions [based on modified Response Evaluation Criteria In Solid Tumors (mRECIST) assessment] [17], Barcelona Clinic Liver Cancer (BCLC) stage B or C categorization, Child–Pugh A classification, Eastern Cooperative Oncology Group performance status (ECOG PS) of 0 or 1, and systolic/diastolic blood pressure < 150/90 mmHg. Moreover, eligible patients were to have adequate bone marrow, hepatic, renal, and pancreatic function. Adequate hepatic function was defined as albumin level of ≥ 2.8 g/dL, bilirubin level of ≤ 3.0 mg/dL, and aspartate aminotransferase, alkaline phosphatase, and alanine aminotransferase levels of ≤ 5 times the upper limit of normal [16]. Patients were excluded if they had previous systemic therapy for HCC and ≥ 50% liver occupation, bile duct invasion, or portal vein invasion at the main portal branch (Vp4). Patient stratification and treatment allocation were based on region (Asia–Pacific or Western); macroscopic portal vein invasion, extrahepatic spread, or both (yes or no); ECOG PS (0 or 1); and body weight (< 60 kg or ≥ 60 kg).

All patients in the original clinical study provided written informed consent. The study protocol, protocol amendments, and informed consent forms were reviewed and approved by the relevant institutional review boards/independent ethics committees. This study was conducted in accordance with the principles of the Declaration of Helsinki and Good Clinical Practice Guidelines.

### Endpoints and clinical assessments

The primary endpoint in REFLECT was OS [16]. The secondary efficacy endpoints included progression-free survival (PFS) and objective response rate (ORR) according to investigator-assessed mRECIST, time to progression according to investigator-assessed mRECIST, and lenvatinib pharmacokinetic exposure parameters. Tumor measurements were performed every 8 weeks using computed tomography or magnetic resonance imaging, regardless of dose interruptions, and until radiologic disease progression [16].

Findings for the primary and secondary endpoints and safety assessments in REFLECT have been published [16]. In this analysis, we assessed OS, PFS, ORR, time to Child–Pugh score ≥ 7, and safety according to patients’ body weights at baseline. Child–Pugh scores were assessed at baseline, and then at the onset of each 28-day treatment cycle.

### Statistical analysis

OS was measured from the date of randomization until the date of death from any cause. Patients who were lost to follow-up were censored at the last date that the patient was known to be alive, and patients who remained alive were censored at the time of data cutoff. The median OS was calculated for each treatment group and presented with two-sided 95% confidence intervals (CIs). Kaplan–Meier estimates of OS for each group were plotted over time. The hazard ratio (HR) and corresponding CIs were calculated using a Cox proportional hazard model and stratified by the randomization stratification factors and treatment group as a factor. PFS was defined as the time from the date of randomization to the date of first documentation of disease progression or the date of death, whichever occurred first; and was assessed using a stratified log-rank test with randomization stratification factors, with the associated HR and 95% CI. Median PFS values are presented with corresponding 2-sided 95% CIs. ORR was determined using mRECIST as assessed by the investigator, and statistical differences between treatment groups were analyzed using the Cochran–Mantel–Haenszel chi-square test with randomization stratification factors as strata, tested at a 2-sided alpha level of 0.05. The 2-sided 95% CIs for the odds ratios (ORs) were calculated. Median time to dose reduction was generated based only on patients who received a reduction. The 95% CI was constructed with a generalized Brookmeyer and Crowley method. REFLECT pharmacokinetic analyses included determining the median and range of individual AUCs at a steady state [16].

## Results

### Patient characteristics

Of the 954 patients randomly assigned in REFLECT, 951 received treatment (lenvatinib *n* = 476; sorafenib *n* = 475) [16]. As previously reported, baseline patient characteristics were similar between the lenvatinib and sorafenib treatment groups, except for baseline hepatitis C etiology and α-fetoprotein concentrations (Table 1) [16]. Patients were predominantly male and approximately two-thirds came from the Asia–Pacific region. Of patients receiving lenvatinib, 151 patients were in the lower body-weight group (< 60 kg) and 325 patients were in the higher body-weight group (≥ 60 kg). Baseline characteristics for patients in the lower and higher body-weight groups (lenvatinib treatment only) were generally similar, except for baseline hepatitis C etiology, region, and sex: while 26.5% of patients in the lower body-weight group had an etiology of hepatitis C virus infection, fewer (15.4%) patients in the higher body-weight group had this etiology; when segregated by region, 86.1% of the patients from the lower body-weight group and 58.8% of patients in the higher body-weight group were from the Asia–Pacific region; and a higher percentage of women was recorded in the lower body-weight group (29.8%) compared with the higher body-weight group (8.6%), although the overall percentage of women in this study was small (15.3%).

**Table 1:** Baseline patient characteristics in REFLECT [16]

Characteristic	Lenvatinib 8 mg (*n* = 151)	Lenvatinib 12 mg (*n* = 325)	Lenvatinib, overall (*n* = 478)	Sorafenib 800 mg (*n* = 476)
Median age, years (range)	65.0 (20–86)	62.0 (24–88)	63.0 (20–88)	62.0 (22–88)
Sex, *n* (%)				
Male	106 (70.2)	297 (91.4)	405 (84.7)	401 (84.2)
Female	45 (29.8)	28 (8.6)	73 (15.3)	75 (15.8)
Region^a^, *n* (%)				
Western	21 (13.9)	134 (41.2)	157 (32.8)	157 (33.0)
Asia–Pacific	130 (86.1)	191 (58.8)	321 (67.2)	319 (67.2)
Bodyweight, *n* (%)				
< 60 kg	151 (100.0)	2 (0.6)	153 (32.0)	146 (30.7)
≥ 60 kg	0	323 (99.4)	325 (68.0)	330 (69.5)
ECOG PS, *n* (%)				
0	93 (61.6)	211 (64.9)	304 (63.6)	301 (63.4)
1	58 (38.4)	114 (35.1)	174 (36.4)	175 (36.8)
Child–Pugh class, *n* (%)				
A	151 (100.0)	322 (99.1)	475 (99.4)	471 (99.0)
B	0	3 (0.9)	3 (0.6)	5 (1.0)
Macroscopic portal vein invasion, extrahepatic spread, or both, *n* (%)				
Yes	105 (69.5)	223 (68.6)	329 (68.8)	336 (70.6)
No	46 (30.5)	102 (31.4)	149 (31.2)	140 (29.4)
BCLC stage, *n* (%)				
B	32 (21.2)	71 (21.8)	104 (21.8)	92 (19.3)
C	119 (78.8)	254 (78.2)	374 (78.2)	384 (80.7)
Etiology^b^, *n* (%)				
Hepatitis B virus	83 (55.0)	167 (51.4)	251 (52.5)	228 (47.9)
Hepatitis C virus	40 (26.5)	50 (15.4)	91 (19.0)	126 (26.5)
Alcohol	6 (4.0)	30 (9.2)	36 (7.5)	21 (4.4)
Other	8 (5.3)	30 (9.2)	38 (7.9)	32 (6.7)
Unknown	14 (9.3)	48 (14.8)	62 (13.0)	69 (14.5)
Baseline α-fetoprotein concentration, *n* (%)				
< 200 ng/mL	80 (53.0)	174 (53.5)	255 (53.3)	286 (60.1)
≥ 200 ng/mL	71 (47.0)	150 (46.2)	222 (46.4)	187 (39.3)
Missing	0	1 (0.3)	1 (0.2)	3 (0.6)

This table includes data both from patients randomly assigned to study drugs and patients treated with study drugs. 478 Patients were randomly assigned to receive lenvatinib but only 476 received treatment. 476 patients were randomly assigned to receive sorafenib but only 475 received treatment

*BCLC* Barcelona Clinic Liver Cancer, *ECOG PS* Eastern Cooperative Oncology Group performance status

^a^“Western region” consists of North America and Europe and also includes Russia and Israel; “Asia–Pacific region” consists of China, Hong Kong, Japan, Korea, Malaysia, Philippines, Singapore, Taiwan, and Thailand

^b^Based on the combined data from HCC diagnosis and medical history. Patients may be counted in more than one category

### Efficacy

**Fig. 1:**
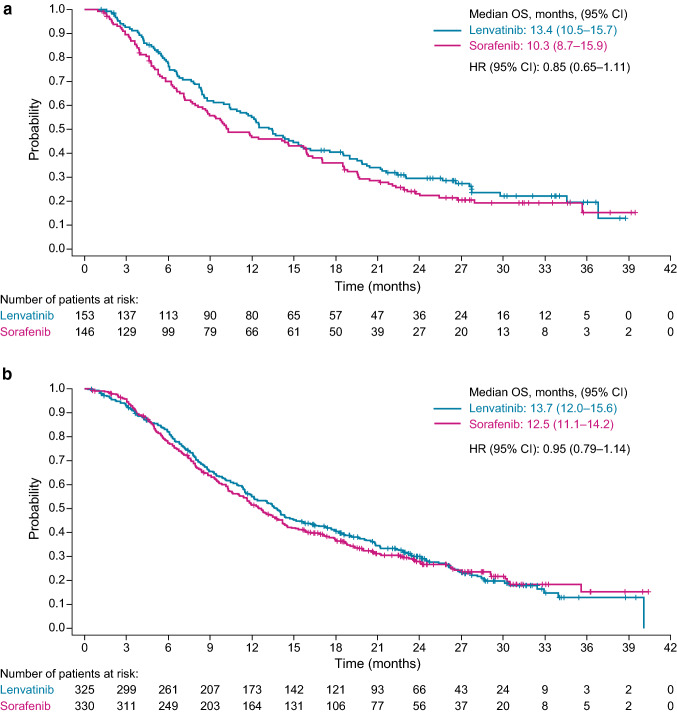
Kaplan–Meier estimates of overall survival in REFLECT, stratified by patient body weight (**a** < 60 kg; **b** ≥ 60 kg). **a** body weight < 60 kg; lenvatinib dose 8 mg/kg. **b** body weight ≥ 60 kg; lenvatinib dose 12 mg/kg

**Fig. 2:**
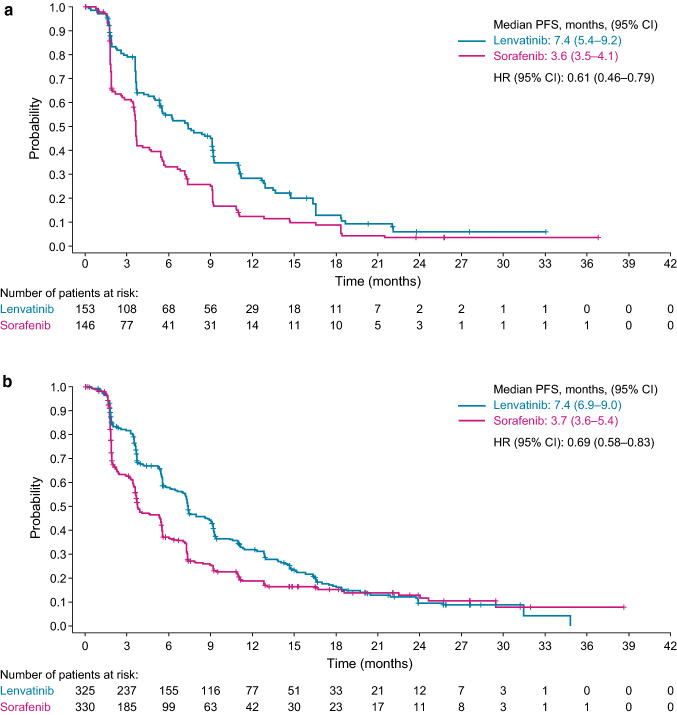
Kaplan–Meier estimates of progression-free survival in REFLECT, stratified by patient body weight (**a** < 60 kg; **b** ≥ 60 kg). **a** Body weight < 60 kg; lenvatinib dose 8 mg/kg. **b** Body weight ≥ 60 kg; lenvatinib dose 12 mg/kg

OS outcomes from REFLECT were comparable between both lenvatinib starting dose groups and OS outcomes were comparable for sorafenib, irrespective of patient body-weight. The median OS duration for patients who received lenvatinib in the lower body-weight group was 13.4 months (95% CI 10.5–15.7) and was 10.3 months (95% CI 8.7–15.9) among patients who received sorafenib (HR 0.85, 95% CI 0.65–1.11). For patients in the higher body-weight group who received lenvatinib the median OS was 13.7 months (95% CI 12.0–15.6), and 12.5 months (95% CI 11.1–14.2) for patients who received sorafenib (HR 0.95, 95% CI 0.79–1.14) (Fig. 1, Supplemental Table 1). PFS outcomes were also comparable between lenvatinib starting dose groups. Patients in both lenvatinib groups had a PFS duration of 7.4 months as measured by mRECIST per investigator assessment (lower body-weight group, 95% CI 5.4–9.2; higher body-weight group, 95% CI 6.9–9.0) (Fig. 2, Supplemental Table 1).

ORRs, as measured by mRECIST per investigator assessment, were also similar between lenvatinib treatment groups: for patients in the lenvatinib lower body-weight group, the ORR was 22.2% (95% CI 15.6–28.8) and the ORR for patients in the lenvatinib higher body-weight group was 24.9% (95% CI 20.2–29.6) (Supplemental Table 1).

Pharmacokinetic exposures for the median values and ranges of AUC were similar between both lenvatinib body-weight groups in REFLECT. In the lower body-weight group, the median was 1820.2 ng × h/mL (range 704.8–4980.7) and in the higher body-weight group, the median was 1996.0 ng × h/mL (range 925.5–5427.9) [16].

### Safety

Rates of exposure to the study-drug were similar between the two lenvatinib groups (Table 2). The median duration of treatment was 5.6 months (range 0.1–33.7) in the lenvatinib lower body-weight group and 6.3 months (range 0–35.0) in the lenvatinib higher body-weight group. Planned starting dose was achieved in a mean of 87.7% (standard deviation 19.84; median 100%) of patients in the lenvatinib lower body-weight group, and in a mean of 87.5% (standard deviation 54.53; median 96.0%) of patients in the lenvatinib higher body-weight group. Mean dose intensities measured 8.0 mg/day/patient (range 2.1–8.0) in the lenvatinib lower body-weight group, and 11.5 mg/day/patient (range 1.7–120.0) the lenvatinib higher body-weight group (Table 2).

**Table 2:** Study-drug exposure and dose modifications due to TEAEs

Parameter	Lenvatinib 8 mg (*n* = 151)	Lenvatinib 12 mg (*n* = 325)	Lenvatinib, overall (*n* = 476)
Median duration of treatment^a^, months (range)	5.6 (0.1–33.7)	6.3 (0–35.0)	5.7 (0–35.0)
Median dose intensity, mg/day/patient (range)	8.0 (2.1–8.0)	11.5 (1.7–120.0)^b^	8.9 (1.7–120.0)
Mean % of planned starting dose received^c^ (standard deviation)	87.7 (19.84)	87.5 (54.53)	87.5 (46.40)
Patients with treatment-related^d^ TEAEs leading to dose modifications, *n*^e^ (%)^f^			
Study-drug discontinuation	16 (10.6)	26 (8.0)	42 (8.8)
Study-drug dose reduction	42 (27.8)	134 (41.2)	176 (37.0)
Study-drug interruption	55 (36.4)	135 (41.5)	190 (39.9)
Study-drug dose reduction or interruption	65 (43.0)	187 (57.5)	252 (52.9)

This table includes only patients treated with lenvatinib (*n* = 476), not the entire randomized data set (*n* = 478)

*TEAE* treatment-emergent adverse event

^a^Duration of treatment (in months) = (date of last dose of study drug − date of first dose of study drug + 1) ÷ 30.4375

^b^One patient took a single dose of lenvatinib 120 mg and was subsequently removed from the study

^c^Defined as the actual dose received as a percentage of planned starting dose (without interruption or reduction). Calculated as cumulative total dose divided by (planned starting daily dose × treatment duration in days)

^d^Related TEAEs include TEAEs that were considered by the investigator to be possibly or probably related to study drug, or TEAEs with a missing causality

^e^Patients may be counted in more than one subcategory

^f^Percentages are based on the total number of patients in the safety analysis set within the relevant treatment group

Study discontinuation of lenvatinib because of treatment-related TEAEs was reported for 10.6% of patients in the lenvatinib lower body-weight group and 8.0% of patients in the lenvatinib higher body-weight group (Table 2). Among patients in the lenvatinib lower body-weight group, treatment-related TEAEs led to dose reduction or interruption in 43.0% of patients. In the lenvatinib higher body-weight group, treatment-related TEAEs resulted in dose reduction or interruption in 57.5% of patients (Table 2). Among patients in either body-weight group who experienced a lenvatinib dose reduction, the median time to first dose reduction was 10.0 weeks (interquartile range 3.4–22.3).

The five most common any-grade TEAEs (lower/higher body weight group) associated with lenvatinib treatment were hypertension (43.0%/41.8%), diarrhea (35.1%/40.3%), decreased appetite (33.1%/34.5%), decreased weight (28.5%/32.0%), and fatigue (27.8%/30.5%) (Table 3).

**Table 3:** Most common TEAEs (> 15% in either lenvatinib group) in REFLECT—provided both by patient incidence (*n*, %) and by TEAE rate (episodes/patient-year of treatment)

Preferred term	Lenvatinib 8 mg (*n* = 151)* n* (%)^a^	Lenvatinib 12 mg (*n* = 325)* n* (%)^a^	Lenvatinib, overall (*n* = 476)* n* (%)^a^	Lenvatinib 8 mg (*n* = 151) TEAE rate (episodes/P-Y)^b^	Lenvatinib 12 mg (*n* = 325) TEAE rate (episodes/P-Y)^b^	Lenvatinib, overall (*n* = 476) TEAE rate (episodes/P-Y)^b^
Any TEAE	151 (100.0)	319 (98.2)	470 (98.7)	18.26	19.15	18.89
Hypertension	65 (43.0)	136 (41.8)	201 (42.2)	0.79	0.78	0.78
Diarrhea	53 (35.1)	131 (40.3)	184 (38.7)	1.06	0.99	1.01
Decreased appetite	50 (33.1)	112 (34.5)	162 (34.0)	0.63	0.59	0.60
Decreased weight	43 (28.5)	104 (32.0)	147 (30.9)	0.50	0.51	0.51
Fatigue	42 (27.8)	99 (30.5)	141 (29.6)	0.52	0.47	0.48
Proteinuria	37 (24.5)	80 (24.6)	117 (24.6)	0.56	0.48	0.50
PPES	35 (23.2)	93 (28.6)	128 (26.9)	0.38	0.46	0.44
Dysphonia	28 (18.5)	85 (26.2)	113 (23.7)	0.30	0.45	0.40
Decreased platelet count	26 (17.2)	61 (18.8)	87 (18.3)	0.35	0.37	0.36
Hypothyroidism	25 (16.6)	53 (16.3)	78 (16.4)	0.26	0.24	0.24
Nausea	24 (15.9)	69 (21.2)	93 (19.5)	0.32	0.36	0.35
Pyrexia	24 (15.9)	45 (13.8)	69 (14.5)	0.29	0.23	0.25
Increased blood bilirubin	23 (15.2)	48 (14.8)	71 (14.9)	0.33	0.29	0.30
Peripheral edema	23 (15.2)	43 (13.2)	66 (13.9)	0.24	0.27	0.26
Vomiting	22 (14.6)	55 (16.9)	77 (16.2)	0.32	0.33	0.33
Abdominal pain	19 (12.6)	62 (19.1)	81 (17.0)	0.22	0.38	0.33
Constipation	19 (12.6)	57 (17.5)	76 (16.0)	0.21	0.30	0.27

This table includes only patients treated with lenvatinib (*n* = 476), not the entire randomized data set (*n* = 478). For patient incidence (%), patients with two or more TEAEs in the same preferred term were counted only once; for TEAE rates, multiple episodes of the same adverse event in each patient were all counted

*PPES* palmar-plantar erythrodysesthesia syndrome, *P-Y* patient-year; *TEAE* treatment-emergent adverse event

^a^Percentages are based on the total number of patients in the safety analysis set within the relevant treatment group

^b^TEAE rate (episode/patient-year) = total occurrence of TEAE episodes (*n*) divided by total duration in each treatment group

When adjusted by treatment duration, rates of TEAEs were slightly lower in the lenvatinib lower body weight group (Table 4): overall, TEAE rates were 18.26 episodes per patient-year (EPY) in the lenvatinib lower body weight group and 19.15 EPY in the lenvatinib higher body weight group. For treatment-related TEAEs, patients in the lenvatinib lower body weight group had a rate of 10.24 EPY, and a rate of treatment-related TEAEs of grade ≥ 3 of 1.32 EPY; the rate of serious TEAEs was 1.19 EPY. Patients in the higher body weight lenvatinib group showed a numerically slightly increased rate of overall treatment-related TEAEs (11.23 EPY), as well as numerically slightly increased rates of treatment-related TEAEs grade ≥ 3 (1.71 EPY) and serious TEAEs (1.29 EPY). Patients receiving lenvatinib experienced a fatal TEAE at a rate of 0.19 EPY overall. In the lower body weight group, the rate was 0.15 EPY, and in the higher body weight group, the rate was 0.21 EPY (Table 4).

**Table 4:** Summary of TEAEs, adjusted by treatment duration

Parameter^a^	Lenvatinib 8 mg (*n* = 151; total duration^b^ = 95.1 years) TEAE rate	Lenvatinib 12 mg (*n* = 325; total duration^b^ = 229.1 years) TEAE rate	Lenvatinib, overall (*n* = 476; total duration^b^ = 324.2 years) TEAE rate
Any TEAE episodes, adjusted by P-Y	18.26	19.15	18.89
Related TEAE episodes^c^, adjusted by P-Y	10.24	11.23	10.94
Related TEAEs with worst CTCAE grade of ≥ 3, adjusted by P-Y	1.32	1.71	1.59
Any serious TEAE episodes, adjusted by P-Y	1.19	1.29	1.26
Fatal TEAE episodes, adjusted by P-Y^d,e^	0.15	0.21	0.19
Nonfatal serious TEAE episodes, adjusted by P-Y	1.14	1.18	1.17

This table includes only patients treated with lenvatinib (*n* = 476), not the entire randomized data set (*n* = 478)

*CTCAE* common terminology criteria for adverse events, *P-Y* patient-year, *TEAE* treatment-emergent adverse event

^a^TEAE rate (episode/P-Y) = total occurrence of TEAE episodes (*n*) divided by total duration in each treatment group

^b^Total duration = sum of treatment time (in years) for all patients in each treatment group (including dose interruptions)

^c^Related TEAEs include TEAEs that were considered by the investigator to be possibly or probably related to study drug, or TEAEs with a missing causality

^d^Patients with fatal TEAEs may also have reported nonfatal serious TEAEs

^e^Fatal TEAE episodes are counted only once per patient, if more than one fatal TEAE was reported for the same patient

**Fig. 3:**
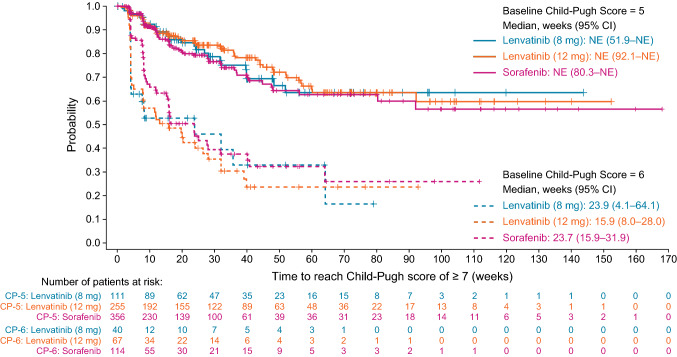
Kaplan–Meier estimates of time to reach a Child–Pugh score of ≥ 7, from baseline score of 5 or 6, following lenvatinib (two dose levels, assigned according to patient body weight) or sorafenib treatment

The effect of lenvatinib starting dose on liver function was analyzed based on the length of time taken to develop a Child–Pugh score of ≥ 7 (Fig. 3) from baseline scores of either 5 or 6. For patients with a baseline Child–Pugh score of 5, the median time (in weeks) to a Child–Pugh score ≥ 7 was not estimable (NE) (95% CI 51.9–NE) in the lenvatinib lower body weight group, and NE (95% CI 92.1–NE) in the lenvatinib higher body weight group. For patients with a baseline Child–Pugh score of 6, the median time to a Child–Pugh score ≥ 7 was 23.9 weeks (95% CI 4.1–64.1) in the lenvatinib lower body weight group, and 15.9 weeks (95% CI 8.0–28.0) in the lenvatinib higher body weight group.

## Discussion

This exploratory post hoc analysis used data from REFLECT to examine the efficacy and safety profiles of lenvatinib when patients were stratified by a body-weight-adjusted starting dose (ie, patients weighing < 60 kg received lenvatinib 8 mg/day; those weighing ≥ 60 kg received lenvatinib 12 mg/day). The results indicate that the efficacy of lenvatinib, as demonstrated by OS, PFS, and ORR outcomes, was comparable between the two body weight groups. Additionally, body weight did not appear to impact sorafenib efficacy. The safety profiles among patients who received lenvatinib were also comparable, with generally similar TEAE rates and types between the body weight groups. This confirms that the body weight-based dosing regimen for lenvatinib is appropriate for this indication, as there was no loss in efficacy with the lower dose.

Prior to this analysis, the impact of body weight on lenvatinib pharmacokinetics and safety was analyzed in patients with HCC in Study 202 (*N* = 46) [13]. While efficacy results were positive in Study 202 (median OS of 18.7 months), all patients exhibited at least 1 TEAE [13]. The most common TEAEs were hypertension (76%), palmar-plantar erythrodysesthesia syndrome (65%), decreased appetite (61%), and proteinuria (61%). Within Study 202, a high percentage of patients (74%) required a dose reduction, and 22% of patients discontinued lenvatinib treatment due to adverse events. Furthermore, 48% of patients experienced a dose reduction or discontinuation as a result of a TEAE < 30 days after the onset of lenvatinib treatment [13].

Further exploration of this situation suggested that low body weight could be a risk factor for early study-drug dose reduction, which led to a pharmacokinetic analysis in patients with HCC to determine the optimal dose of lenvatinib [15]. This analysis revealed that when dosing was adjusted for body weight (patients weighing < 60 kg received a starting dose of 8 mg/day lenvatinib, while patients weighing ≥ 60 kg received a starting dose of 12 mg/day lenvatinib), the AUC between the two groups was similar. Using data from Study 202, correlations between rates of dose reduction or discontinuation and factors including body weight and body surface area were assessed. Ultimately the results of Study 202 [13] and the pharmacokinetic data [15] led to the decision to implement body weight-based dosing in REFLECT due to its clinical versatility. The goal of body weight-based dosing was to maintain efficacy while decreasing study-drug-related adverse events.

In support of the use of body weight-based lenvatinib dosing in patients with uHCC, efficacy and safety findings in REFLECT were similar between the two lenvatinib dosing groups. The primary endpoint of this analysis of data from REFLECT was OS, and median OS durations were similar in both lenvatinib groups (13.4 months in the lower body weight group, 13.7 months in the higher body weight group) (Fig. 1). Additionally, the TEAE profiles were also similar between the lenvatinib groups, with the five most common types of events in each group being hypertension, diarrhea, decreased appetite, decreased weight, and fatigue (Table 3). In REFLECT, 27.8% of patients in the lenvatinib lower body weight group and 41.2% of patients in the lenvatinib higher body weight group underwent dose reductions (Table 2). Treatment-related TEAEs led to lenvatinib discontinuation in 10.6% of patients in the lower body weight group and 8.0% in the higher body weight group. When the higher body weight group of REFLECT was analyzed further in a post hoc analysis specifically examining patients with body weights > 80 kg, the efficacy (PFS by investigator-assessed mRECIST 9.2 months; OS 14.9 months) and safety profiles of lenvatinib in this group were comparable to those of patients within the current study [18].

While data from Study 202 were used to support the lenvatinib dose regimen utilized in REFLECT, there are several key differences in baseline patient characteristics. REFLECT was a phase 3 study with most patients (*n* = 476 patients who received lenvatinib) coming from two regions (Table 1). Approximately two-thirds of all patients were from the Asia–Pacific region. In contrast, all patients in Study 202 were from either Japan (*n* = 43) or South Korea (*n* = 3) [13]. Most patients from Study 202 had an etiology of hepatitis B (35%) or C (45%), and 15% had an etiology of alcohol; one patient (5%) had an unknown etiology [13]. Within the lenvatinib arm of REFLECT, baseline characteristics were similar between the lenvatinib dosing groups, including measurements of disease severity such as ECOG PS and BCLC. Among all patients receiving lenvatinib, approximately 60% had an ECOG PS of 0, while approximately 80% of patients were assessed as having a BCLC rating of stage C. However, in Study 202, 83% of patients had an ECOG PS of 0, and 59% had a BCLC rating of stage C [13]. Finally, patients were excluded from REFLECT if they had received prior systemic therapy for HCC, whereas in Study 202 some patients had received prior chemotherapy (13% had received sorafenib, 11% had received other systemic chemotherapy, and 11% had received hepatic intra-arterial chemotherapy [13]).

Discussion regarding oncology treatment dose selection has indicated that dosing assessment should be an ongoing process, with adjustments made based on indications and special populations [19]. Pharmacokinetic evaluation is particularly valuable in these assessments because it can determine the balance between peak efficacy and adverse events [19]. The pharmacokinetic analysis conducted to determine the optimal lenvatinib dose regimen for patients with uHCC used in REFLECT is one such example of the need for, and effectiveness of, these assessments [15].

To our knowledge, REFLECT was the first phase 3 trial of patients with advanced HCC that utilized body weight-based dosing with a multikinase inhibitor [16] and, thus could provide valuable information for future clinical trials and study-drug administration procedures. In addition to the pharmacokinetic analysis demonstrating comparable lenvatinib AUC between the two body weight groups [16], the similar efficacy and safety results between the two body weight groups presented in this post hoc analysis of data from REFLECT support the use of a body weight-adjusted lenvatinib dosing regimen for patients with uHCC.

## Supplementary Information

Below is the link to the electronic supplementary material.

Supplementary file1 (DOCX 46 KB)
