# Supplementary material for: Safety and efficacy of lenvatinib by starting dose based on body weight in patients with unresectable hepatocellular carcinoma in REFLECT
Source: J Gastroenterol. 2021 May 4;56(6):570–80. doi: 10.1007/s00535-021-01785-0 (PMC8137475; doi:10.1007/s00535-021-01785-0)
Supplement: Supplementary file 1 — Supplementary file1 (DOCX 46 KB) [file 535_2021_1785_MOESM1_ESM.docx]

**Supplementary Material**

**Safety and efficacy of lenvatinib by starting dose based on bodyweight in patients with unresectable hepatocellular carcinoma in REFLECT**

**Authors:** Takuji Okusaka^1^, Kenji Ikeda^2^, Masatoshi Kudo^3^, Richard S. Finn^4^, Shukui Qin^5^, Kwang-Hyub Han^6^, Ann-Lii Cheng^7^, Fabio Piscaglia^8^, Masahiro Kobayashi^2^, Max W. Sung^9^, Minshan Chen^10^, Lucjan Wyrwicz^11^, Jung-Hwan Yoon^12^, Zhenggang Ren^13^, Kalgi Mody^14^, Corina E. Dutcus^14^, Toshiyuki Tamai^15^, Min Ren^14^, Seiichi Hayato^15^, Hiromitsu Kumada^2^

**Author affiliations**: ^1^National Cancer Center Hospital, Tokyo, Japan; ^2^Toranomon Hospital, Tokyo, Japan; ^3^Kindai University Faculty of Medicine, Osaka, Japan; ^4^Geffen School of Medicine, UCLA Medical Center, Santa Monica, CA, USA; ^5^Nanjing Bayi Hospital, Nanjing, Jiangsu, China; ^6^Severance Hospital, Yonsei University, Seoul, Korea; ^7^National Taiwan University Hospital, Taipei, Taiwan; ^8^Azienda Ospedaliero Universitaria S. Orsola Malpighi di Bologna, Bologna, Italy; ^9^Tisch Cancer Institute at Mount Sinai, New York, NY, USA; ^10^Sun Yat-sen University Cancer Center, Guangzhou, China; ^11^Narodowy Instytut Onkologii, Warsaw, Poland; ^12^Seoul National University Hospital, Seoul, Republic of Korea; ^13^Zhongshan Hospital Fudan University, Shanghai, China; ^14^Eisai Inc., Woodcliff Lake, NJ, USA; ^15^Eisai Co. Ltd, Tokyo, Japan

**Corresponding author:** Takuji Okusaka, National Cancer Center Hospital, Tokyo, Japan, tokusaka@ncc.go.jp

**Supplemental Table 1. Efficacy responses in patients randomly assigned to receive lenvatinib (then stratified by bodyweight) per mRECIST by investigator assessment^a^**

| **Efficacy Parameter** | **Lenvatinib 8 mg**  **(n = 153)** | **Lenvatinib 12 mg**  **(n = 325)** |
| --- | --- | --- |
| **Overall survival, months (95% CI)** | 13.4  (10.5–15.7) | 13.7  (12.0–15.6) |
| **Progression-free survival, months (95% CI)** | 7.4  (5.4–9.2) | 7.4  (6.9–9.0) |
| **Objective response rate, % (95% CI)** | 22.2  (15.6–28.8) | 24.9  (20.2–29.6) |

^a^This table includes all patients randomly assigned to lenvatinib.

CI, confidence interval; mRECIST, modified Response Evaluation Criteria In Solid Tumors.
